# Six-Month Home-Based Telemedicine Program for Heart Failure and Type 2 Diabetes Patients: Applicability, Usability of Telemonitoring Devices and Apps, and Patient Satisfaction

**DOI:** 10.3390/healthcare14010090

**Published:** 2025-12-30

**Authors:** Palmira Bernocchi, Gloria Fiorini Aloisi, Marilisa Serlini, Elisa Pasotti, Laura Comini, Simonetta Scalvini

**Affiliations:** 1 Continuity of Care Service, Institute of Lumezzane, Istituti Clinici Scientifici Maugeri IRCCS, 25065 Lumezzane, Italyisa.serlini@icsmaugeri.it (M.S.); elisa.pasotti@icsmaugeri.it (E.P.); simonetta.scalvini@icsmaugeri.it (S.S.); 2 Scientific Direction of the Institute of Lumezzane, Istituti Clinici Scientifici Maugeri IRCCS, 25065 Lumezzane, Italy; laura.comini@icsmaugeri.it

**Keywords:** teleassistance, telemonitoring, mobile phone apps, mHealth, digital health, chronic disease, system usability scale

## Abstract

**Background:** Telemedicine can improve early symptom detection using medical devices and applications. It can also help identify barriers to patient adherence and enhance communication with healthcare professionals. This study aimed to evaluate the applicability, usability, and patient satisfaction with telemonitoring devices and apps for individuals with heart failure and type 2 diabetes. **Methods:** In a randomized study, patients in the Intervention Group received six months of nursing teleassistance and telemonitoring using a wearable electrocardiograph, a step tracker, and an App for recording clinical information and conducting video calls. Usability was measured using the System Usability Scale (SUS) and satisfaction with a six-item questionnaire. **Results:** A total of 43 patients (71 ± 8 years) were enrolled in the intervention group. A total of 41 (95%) of patients utilized the App daily, entering 13,048 information, 53 ± 59 per patient. The nurses performed 896 video-calls, 22 ± 21 per patient. The mean number of walking sessions recorded was 6.1 ± 0.9 per week (159 ± 24 per patient). Thirty-five patients (81%) used a 3-lead ECG and recorded 942 traces, 27 ± 14 per patient. At the end, 40 SUS were collected from patients: 15 (38%, 71 ± 7 years) considered the system excellent or good, 20 (50%, 71 ± 8 years) thought it fair, and 5 (13%, 74 ± 7 years) considered the system offered poor. The overall assessment of patient satisfaction with the service was 22 ± 3.3. **Conclusions:** This study provides evidence that, although technology can be complex for older adults, it is broadly accepted by most patients, especially when the benefits are understood. The support offered by nurses is essential for significantly enhancing the overall patient experience.

## 1. Introduction

Heart failure and type 2 diabetes are critical public health challenges affecting millions of individuals across Europe, contributing to increasing morbidity and healthcare costs [1,2]. Chronic patients grappling with these cardiovascular and metabolic disorders must adopt specific daily self-management behaviors to stabilize their health and enhance their quality of life effectively. Key practices include consistently monitoring vital signs such as blood pressure and blood glucose levels, participating in regular physical activities tailored to their capabilities, and adhering to a nutritious, well-balanced diet that supports overall health [3,4,5]. Optimized therapy—encompassing appropriate medication management, dietary adjustments, and lifestyle changes—combined with self-monitoring and a commitment to a healthy lifestyle, can significantly slow the progression of heart failure and type 2 diabetes. These strategies can help alleviate symptoms, reduce the incidence of acute episodes, and minimize the frequency of hospitalizations, ultimately improving patient outcomes [2].

Telemedicine has emerged as a promising strategy to improve care for chronic conditions such as heart failure (HF) and type 2 diabetes (T2D), where continuous monitoring and patient engagement are essential [6]. Yet, evidence on long-term sustainability, cost-effectiveness, and broader outcomes, such as quality of life, remains limited. Studies also report barriers related to digital literacy, patient engagement, and standardization of intervention components [7].

The advent of mobile health (mHealth) devices has offered innovative solutions to support self-management in patients [5,8]. Over the past decade, there has been a substantial increase in the availability and functionality of home telemonitoring devices and mobile health applications. These technologies provide patients with tools to track their health metrics in real time, receive reminders for medication, and access educational resources, empowering them to manage their conditions more effectively [5,8].

Teleassistance and telemonitoring represent promising methods for providing ongoing support and monitoring to patients with chronic conditions within a comprehensive, integrated care model. These approaches enable healthcare providers to detect symptoms early by leveraging medical devices and applications that continuously monitor patient data. Furthermore, they can identify barriers to treatment adherence, assess the effectiveness and intensity of physical therapy, and enhance communication among healthcare professionals involved in a patient’s care [8,9,10].

Teamwork and collaboration are essential for ensuring the highest quality of care and improving patients’ awareness of their health status [11,12]. Actively engaging patients in managing their chronic diseases is crucial for enhancing their self-management skills and overall quality of life.

Although some studies demonstrate that patients find using mobile devices for self-management feasible and helpful [13,14,15], several challenges can hinder their effective utilization. Common obstacles include technological difficulties, a sense of work overload, and a lack of comprehension regarding the importance of the information collected through these devices, which may lead to discontinuation of their use [16,17]. Given these challenges, conducting comprehensive usability analyses is crucial for identifying factors that affect patient satisfaction and efficacy. Understanding specific usability issues—such as the intuitiveness of the user interface, accessibility of features, and the relevance of collected data—can help healthcare professionals implement necessary modifications to improve monitoring processes and enhance the overall patient experience.

Older patients with chronic conditions often face barriers to adopting technology-based interventions, including usability challenges, digital literacy issues, and physical or cognitive limitations. These factors can significantly impact adherence and the effectiveness of teleassistance programs. Despite the growing use of telemedicine, there is limited evidence on the usability of device-based teleassistance specifically in this population. Our study addresses this gap in a mixed phenotype disease (both CHF and diabetes) by providing preliminary data on user experience and feasibility, which are essential for designing interventions that are both practical and acceptable in real-world settings.

A well-established tool for assessing the usability of digital products is the System Usability Scale (SUS), which has been widely utilized since its introduction in 1986 [18]. A scoping review [19] highlighted that the SUS remains the most commonly employed questionnaire for evaluating usability across various digital health technologies.

Overall, existing literature highlights the need for research that addresses these gaps by focusing on usability, patient experience, and integration of technology into routine care [20,21].

This study aims to thoroughly examine the applicability, usability, and patient satisfaction associated with a range of telemonitoring devices and applications employed within an integrated care pathway specifically designed for individuals with chronic heart failure and type 2 diabetes. By doing so, it seeks to identify critical factors that can inform the future design and implementation of these tools, ultimately improving the management of these chronic conditions.

## 2. Methods

This is an exploratory study only on patients enrolled in the Intervention Group at Istituti Clinici Scientifici Maugeri-Institute of Lumezzane. These patients were included in a randomized controlled trial (RCT) [22] and participated in six months of nursing teleassistance and a telemonitoring program using different devices.

Patients were screened at baseline and re-evaluated six months later during a routine outpatient cardiology visit for chronic heart failure.

In detail, the protocol provided for patients the use of a wearable electrocardiograph three leads (CGM HI 3-leads, CompuGroup Medical SE, Milan, Italy) to register traces during physical activity, the smartwatch Fitbit Inspire 2 to register daily steps and heart rate (https://www.fitbit.com/) and the App TreC Version 1.2 (Fondazione Bruno Kessler, Trento, Italy) to view medication history and upcoming doses. This allows patients to confirm whether they have taken their daily therapy or provide a justification for non-compliance, enter, view, and modify self-detected clinical parameters and symptoms, receive reminders for healthcare actions (e.g., measuring blood pressure), chat, send images/PDFs, and make video calls with healthcare personnel.

In addition, patients were equipped with a data-connected smartphone to send data using the necessary apps for managing the devices, collecting clinical data, as well as facilitating communication with healthcare professionals.

The health personnel used two web platforms to receive data from patients after authentication: the CGM Care Map web portal (CompuGroup Medical SE, CGM, Milan, Italy) and the “TreC” web dashboard (Fondazione Bruno Kessler, Trento, Italy).

The CGM Care Map (Medical Device Class IIa certified) is a platform consisting of a web portal for patient management by healthcare personnel, which acquires vital parameters and data on fitness. Vital parameters are measured by a wearable electrocardiogram (ECG) device with three leads, and the results are transmitted via Bluetooth as electrocardiogram traces to a smartphone. The smartphone app serves as an interface through which the associated ECG can be identified and subsequently transmits the gathered data to the server via the CGM Care Map web platform.

The fitness data recorded by the tracker is automatically transmitted to the Fitbit App and the Fitbit server. The data is then retrieved daily from the CGM Care Map server.

The “TreC” web dashboard facilitates the management of patient treatment plans by healthcare personnel through the remote activation of therapies prescribed by medical specialists, the monitoring of patient data, and the viewing of trends in entered data. Furthermore, they have the capacity to administer questionnaires by means of a chatbot and to communicate with patients via chat and video calls.

### 2.1. Intervention

The patients in the Intervention group were subject to a six-month period of observation, during which they participated in a remote home telemedicine programme. This programme was in addition to the standard care they were already receiving, which included visits to their general practitioner and hospital control on request. The telemedicine programme was characterised by the following:

1. The provision of support by a nursing case manager is facilitated through the implementation of a structured teleassistance programme, encompassing the delivery of telephone or video consultations on a weekly basis.
2. Teleconsultations in the fields of cardiology and diabetes are available at the inception of the programme, and, if required, throughout its duration.
3. The provision of assistance to the trainer is intended to encourage the undertaking of daily physical activity.
4. The process of telemonitoring encompasses the continuous observation of patient vital signs, such as electrocardiographic traces, as well as the tracking of daily steps.

The nurse case manager was responsible for coordinating activities and promoting patient learning of disease self-management techniques to prevent exacerbations, thus playing a central role in all home care continuity interventions and serving as a key interface in the dialogue between patients and specialists.

The educational scheme encompassed family members and proved to be instrumental in the programme’s success.

Patients were permitted to contact their respective nurses during operating hours for issues pertaining to clinical matters, symptoms, or treatment-related concerns.

Patients were encouraged to engage in physical activity at least three times a week, with a view to emphasising the significance of lifestyle modifications and exercise. Furthermore, the participants were requested to wear their Fitbit devices in order to record their daily step counts in a continuous manner.

The research team undertook the responsibility of monitoring the data in a continuous manner, with the aim of ensuring that patients complied with the intervention. If the nurse received signs and symptoms of a worsening clinical condition, he/she contacted the patient to resolve problems with device malfunctions, or in the event of failure to enter information into the App.

**Figure 1:**
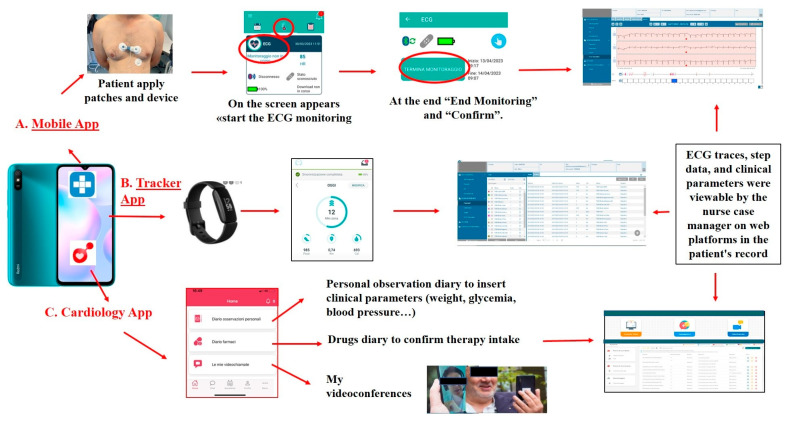
Description of the devices used for the study intervention.

In Figure 1, we describe the devices used for the study.

### 2.2. Usability and Satisfaction Questionnaires

The System Usability Scale (SUS) was utilised to assess the technological system’s efficacy in meeting the needs of the patient cohort undergoing treatment. SUS comprises ten statements (five positive statements and five negative statements, which alternate), each of which is accompanied by a five-point scale ranging from “strongly disagree” to “agree” (Likert ratings) [23,24,25].

In addition to being easy to use and understand for anyone who approaches it, it can be used to evaluate any technology.

The final SUS scores were tallied and scaled to convert the scores to a 0 to 100 scale [26]. The conversion process required subtracting one from each score for odd questions and subtracting the score from 5 for even questions. Then, all the correct scores were added, and the sum was multiplied by 2.5.

Higher scores indicate better usability. Following an “adjective ranting”, scores <50 indicate “difficulty” in usability, more or less important; scores >50 and <70 are considered “good and promising”, and scores of 90 are considered “excellent”.

The goals of SUS, then, were to:

- Give us a measure of people’s subjective perceptions of a system’s usability and
- Allow us to do so in the very short time available during an evaluation session.

The survey was conducted exclusively among patients who returned to the hospital for an outpatient cardiology visit after completing the telemonitoring program. Patients completed the questionnaire independently. Patients were asked to rate only the use of the telemonitoring devices used during the study. After completion, each answer was added to an internal database containing patient information.

Satisfaction was measured at by a questionnaire, with scores from 0 (not at all satisfied) to 4 (very satisfied), that investigates the service as a whole, the use of the devices, the willingness of healthcare professionals, the clarity of indications to the suggestions provided, the feeling of support, and whether the service is perceived as a real help or not.

The survey was conducted exclusively among patients who returned to the hospital for an outpatient cardiology visit after completing the telemonitoring program. Patients completed the questionnaire on their own. Patients were invited to provide a rating solely on the utilization of the telemonitoring devices employed during the course of the study. Subsequent to the completion of each response, it was entered into an internal database that contained patient information.

Satisfaction was measured using a questionnaire with scores ranging from 0 (not at all satisfied) to 4 (very satisfied). The questionnaire investigated the service as a whole, the use of the devices, the willingness of healthcare professionals, the clarity of indications to the suggestions provided, the feeling of support, and whether the service is perceived as a real help or not.

## 3. Results

Between August 2022 and March 2024, 43 chronic complex patients with heart failure and diabetes (mean age 71 ± 8 years, 16% female) were enrolled in the intervention group. Of these, 41 (95%) patients, described in Table 1, with a mean age of 72 ± 8 years, 16% female, used the devices. Out of the 41 patients, 28 (68%) were smartphone users, with a mean age of 72 ± 8 years, while 13 (32%) did not use a personal smartphone, with a mean age of 72 ± 4 years.

Two at home decided not to use the devices, and they were followed exclusively through nurse-led teleassistance (telephone contacts).

Table 2 shows the data relating to the use of the Apps and the devices. Ninety-five percent of patients used the App TreC and FitBit to track daily steps. Eighty-one percent of patients used the CGM App and wearable ECG device to record and send traces.

Over the course of 6 months, we collected critical issues related to device/App usage, which required technical assistance from the dedicated team and the HelpDesks. The results are reported in Table 3.

At the end of the 6-month intervention, we collected data on SUS from 40 patients (93%) who returned for the final outpatient visit. 1 patient didn’t return for the final visit.

**Figure 2:**
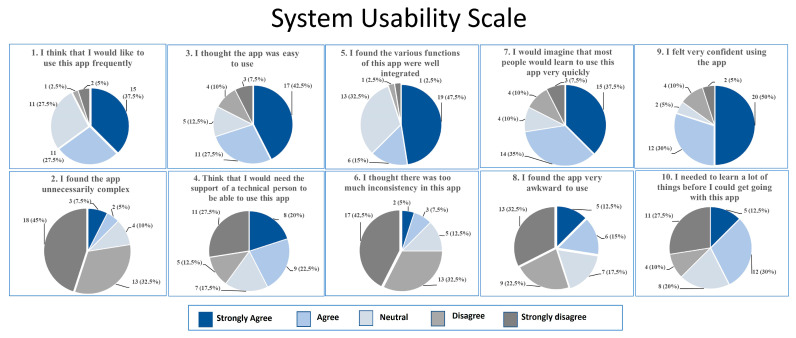
System Usability Scale.

Figure 2 shows the patients’ responses to the ten questions of the scale before the adjustment from the algorithm interpretation.

After the algorithm interpretation, nine (23%) patients considered the system excellent, six (15%) patients considered the experience good, twenty (50%) patients considered the experience fair, and five (13%) patients considered the experience poor.

**Figure 3:**
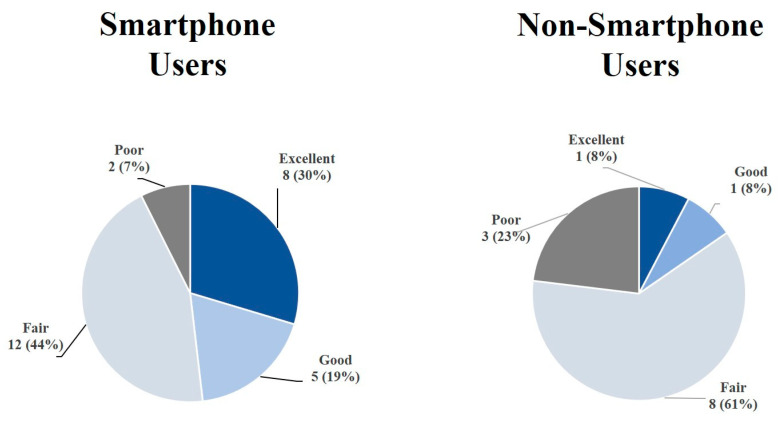
System Usability Scale groups in smartphone and non-smartphone user patients.

In Figure 3, we present the SUS replies from patients, categorized into smartphone users and non-users. The two groups had statistically different behaviors, with a *p*-value of 0.0284 (chi-square analysis).

The median overall patient satisfaction score was 22 (1st–3rd quartiles:19–23), indicating a rating between ‘fairly satisfied’ and ‘very satisfied’. The median of all responses to the six items was 4.0 (1st–3rd quartiles: 3.0–4.0). Table 4 details the single questions reported in the satisfaction questionnaire and the related scores (expressed as median and 1st–3rd quartiles).

**Figure 4:**
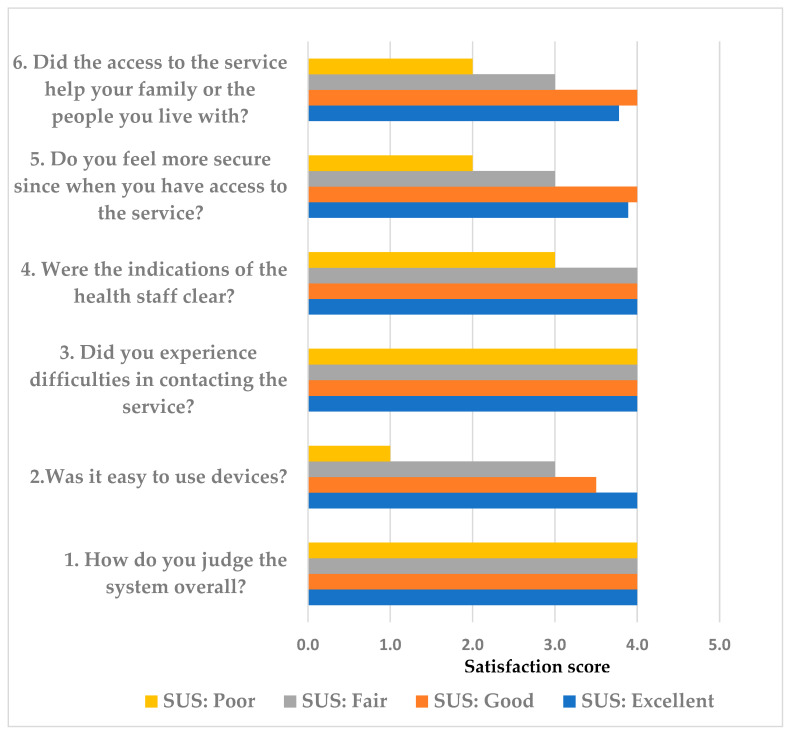
Association between the responses (median values) to each question of the satisfaction questionnaire [Likert scale: 0 (not at all satisfied) to 4 (very satisfied)] for all patients (black bars) and stratified in relation to the four subgroups from the SUS questionnaire.

In Figure 4, we associated the responses to six items of the satisfaction questionnaire with the four subgroups of users resulting from the SUS questionnaire.

Overall, patients who rated SUS as poor scored lower on the satisfaction questionnaire (median value 14), while those who rated SUS as good/excellent scored higher (median values 23.5 and 24, respectively). Patients in the fair group had a mean satisfaction questionnaire score of 20.5, indicating a “fairly satisfied” rating.

## 4. Discussion

This study analysis provides evidence of the applicability and usability of a telemonitoring system in an integrated home-care program for patients with heart failure and type 2 diabetes.

Our results indicate that over 90% of participants were able to use wearable digital devices to transmit clinical parameters to healthcare professionals and monitor their daily steps. Despite difficulties related primarily to the patients’ low digital literacy, numerous parameters were transmitted and used by the case manager nurse and trainer to monitor patients throughout their home care journey.

Thirty-eight % of patients assessed the system as excellent or good. Conversely, 13% of patients gave a negative rating, believing the system to be too tricky and unusable. A significant difference in mean age was noted between the two patient groups, although this difference was not statistically significant in the latter group. It is noteworthy that both groups included patients in the ancient (>80 years) and younger (<65 years) age groups. Finally, 50% of patients gave a “neutral” rating of acceptability, indicating neither a negative nor a positive response.

These conflicting results are reported in most previous papers. Torbjornesen et al. [27] found different results in the acceptability and use of an App in patients with type 2 diabetes. The authors describe how some participants became stressed by the App and mobile phones. Lewinsky et al. [28] examined the perceptions of adults who used multiple mHealth devices simultaneously, including a physical tracker, as in our experience, to generate healthcare data. Despite some challenges, they found that type 2 diabetes patients found the system feasible for facilitating self-management. Similar results were found by Evans et al. [29], who reported an adherence rate of 77% among CHF patients using technology. Better adherence rates were achieved in our research, with 95% for patients using the TREC App, 81% for those using the three-lead ECG, and 95% for those using Fitbit. Although the use of smartphones among older adults is increasing, many still own old-generation cell phones that are not suitable for telemonitoring purposes. The mean age of the patients in the current study was high, and most of them were unfamiliar with smartphones and had never used apps or digital devices. For these reasons, we considered providing patients with a pre-configured smartphone for managing their devices. A pre-configured smartphone allows for more efficient technical management without requiring access to the patient’s phone, which may pose a greater risk of privacy violation and patient refusal. This decision could be a possible explanation for the service’s acceptability to many patients.

With regard to telemonitoring, the TIM-HF2 trial demonstrated that patients with CHF expressed high levels of satisfaction and adherence to the system [11]. In this study, the key components were multi-modal telemonitoring, a pre-interventional home visit by HF nurses, daily measurements with four devices, nursing assessments, monthly telephone contact, and close collaboration with physicians. Close contact throughout the entire study period and nurse-based training have been key in achieving high adherence.

In the current study with integrated care follow-up through telemonitoring, the patient’s nursing case manager has a multi-modal approach in line with the abovementioned trial [11] and plays a key role in helping patients with technology. In particular, the nurse favours close support from operators, prompt feedback when needed and virtual services using digital devices, helping to keep patients safe and active in disease management [12,30,31].

The importance of the nurse case manager support was particularly evident in the results obtained by the satisfaction questionnaire, where the overall score for the service remained elevated, even among patients who gave a lower score in the SUS questionnaire regarding the acceptability of technology.

The study team assisted patients, helped them also to manage technological issues effectively, and addressed the potential risk of increasing anxiety and depression, particularly among older and more vulnerable patients, due to a lack of technological knowledge, as reported by De Luca et al. [32].

In any case, the patient can appreciate the importance of teleassistance, conducted by the nurse case manager, who collects information about the status of disease and symptoms, offers advice on diet, lifestyle, and medications, and establishes a connection with the specialist, as well as the use of telemonitoring.

Usability ratings [33,34] generally improved over time, and this is particularly true for CHF patients, suggesting that attitudinal and cognitive barriers that elderly patients sometimes have towards technology can be successfully addressed, thereby increasing use and enhancing patient engagement and adherence [35,36].

However, some authors emphasize the importance of effectively managing technological issues, as poorly handled problems could cause patients to reject the monitoring device [37,38].

Furthermore, technology cannot always be trusted, and previous studies have identified various obstacles to its use. Technological challenges mean that an app, certain devices and a mobile phone could impose an additional burden on self-management as part of integrated care [8].

Utilization of telemonitoring systems in healthcare is expected to increase; investigating the patient’s needs and including them in a co-creation program could meet end-user requirements in terms of content and usability [36]; the lag time in usual care was overcome by the connected digital devices allowing real-time two ways communication between patient and care team; this permits early recognition of instabilization of the disease, an increase in medical adherence to therapy and the guideline-directed medical therapy, avoiding hospitalization and mortality [8].

The results from the SUS questionnaire show that perceived technology usability is not uniform among patients but is distributed across three distinct profiles (Figure 3). The group with high scores (excellent/good) confirms the feasibility and acceptability of the tool in clinical settings, with high satisfaction with the service offered. On the contrary, the group with negative evaluations (poor) suggests the presence of significant barriers, probably linked to individual factors such as poor digital literacy, resistance to change, or limited perception of the benefits and with lower satisfaction, particularly related to the device.

The intermediate group (fair) in both the smartphone and non-smartphone user groups represents a significant portion of the study population and deserves particular attention. Although these patients expressed fairly satisfaction and not explicit rejection, they have not developed full confidence in the technology. This attitude could compromise long-term adherence and reduce the effectiveness of interventions based on digital tools. As already highlighted in our previous work [12], constant support has proven to be a key factor in improving the user experience. However, to consolidate adoption, it is necessary to implement personalized strategies, including targeted training programs (tailored to the patient’s level of technological competence), motivational interventions aimed at reinforcing the perception of clinical and functional benefits, and ongoing technical support, to reduce the risk of abandonment due to operational difficulties.

When comparing our findings with previous studies on telemedicine interventions for chronic conditions, differences in usability and patient compliance become evident. For type 2 diabetes, mobile health applications often report high usability scores, with System Usability Scale (SUS) values frequently exceeding 80 when user-centered design principles are applied [39]. However, barriers such as digital literacy and age-related limitations remain significant [7]. In contrast, interventions for heart failure show greater variability in adherence, ranging from 60% to over 85%, with higher compliance observed in programs combining remote monitoring and telecoaching [40,41]. Our study differs from these models by focusing exclusively on older patients using device-based teleassistance rather than app-based or hybrid solutions. This population is characterized by specific usability challenges, including motor and cognitive limitations, which may explain the lower SUS scores observed compared to younger or more digitally literate cohorts. These findings highlight the importance of tailoring telemedicine technologies to the needs of older adults to improve both usability and adherence.

### 4.1. Limitation

The present study is not without its limitations. Firstly, although the sample size was considered adequate for the purpose of assessing the usability and feasibility of the service, it was not sufficiently powered to evaluate its effectiveness. It is recommended that future research be conducted in order to ascertain the efficacy of the treatment in a larger patient population. Secondly, the duration of the study was comparatively brief, and the duration of adherence to the App over a more extended period remains to be ascertained. Thirdly, it must be noted that the participants in this study may not be representative of the majority of HF patients. This is due to the fact that they may have been more motivated to participate in the study. Fourthly, the psychometric properties of the satisfaction questionnaire used in this study have not yet been validated, and the findings related to patient satisfaction should be considered exploratory.

### 4.2. Future Lines of Research

To further determine usability and feasibility of device-based teleassistance in older patients, future research could explore several directions. For instance, assessing long-term adherence and comfort with device use, identifying cognitive, motor, and sensory factors that influence usability, and evaluating the impact on clinical outcomes and quality of life. It would also be important to investigate barriers such as digital literacy, caregiver involvement, and technical support needs, as well as to examine cost-effectiveness and scalability in geriatric care settings. Comparative studies between device-assisted and non-device-assisted teleassistance could help define optimal strategies for implementation.

## 5. Conclusions

This study provides evidence that, despite the complexity of technology for elderly patients, most patients would be willing to accept it if they and their families fully understand the benefits that it can offer. The proposed questionnaire (SUS) is not confined to the investigation of the usability of the devices; rather, it encompasses an evaluation of the service in its entirety. It is evident that the assistance provided by the case manager is instrumental in optimizing the patient’s experience and enhancing its practicality.

## Figures and Tables

**Table 1:** Overall patient demographics of the involved patients (*n* = 43).

Demographic Variables	Intervention Group *n* = 43
**Age, years**	71 ± 8
**Gender**	
Female	8 (19%)
Male	35 (81%)
**CIRS:**	
Severity Index	2.2 ± 0.4
Comorbidities Index	5.0 ± 1.9
**NYHA class**	
II	18 (42%)
III	25 (58%)
**Most frequent Comorbidities:**	
Hypertension	29 (67%)
Metabolic syndrome	21 (49%)
Renal Insufficiency	9 (21%)
Respiratory Insufficiency	3 (7%)
**Patients who used devices, *n* (%)**	41 (95%)
**Mean age ± SD, years**	72 ± 7
**Ability to use smartphones**	
Smartphone users, *n* (%)	28 (68%)
Mean age ± SD of smartphone users, years	72 ± 9
Female, *n* (%)	8 (20%)
Patients supported by caregivers, *n* (%)	7 (25%)
Mean age ± SD, years	78 ± 9
Non-smartphone users	13 (32)
Mean age ± SD of non-smartphone users, years	72 ± 4
Female	4 (31)
Patients supported by caregivers	6 (46)
Mean age ± SD	74 ± 3

**Table 2:** Data relating to the use of the Apps and the devices.

APP (TREC)		
	Total number	Mean ± SD per patient/six months
Number of patients who used the App (%)	41 (95)	
Information entered by the patient:		
➢ Therapies managed	502	12 ± 3.9
➢ Blood pressure data	3302	81 ± 61
➢ Glycaemia data	2380	58 ± 63
➢ Heart rate data	2423	59 ± 62
➢ Weight data	2622	64 ± 58
➢ SpO_2_ data	1790	44 ± 66
Video conferences with the case manager:		
➢ Meetings	896	22 ± 21
➢ Minute meetings	4915	120 ± 120
**Telemonitoring ECG 3 leads**		
Number of patients who have performed tracings (%)	35 (81)	
Number of tracings sent remotely and reported	942	27 ± 14
Minutes of tracing		334 ± 450
**APP + Tracker FitBit**		
Number of patients who have used FitBit (%)	41 (95)	
Total number of walking records sent by the tracker	6535	159 ± 24
Walking session/week		6 ± 0.94

**Table 3:** Description of criticalities related to devices /App usage.

	Percentage
Difficulty faced by patients interacting with a new system containing unfamiliar technological content.	30%
Low Internet connectivity at the patient’s home. The lack of a stable connection made it difficult to send the detected data.	25%
Disconnection or difficulty in reconnecting between the smartphone and devices	15%
Reports of “Electrodes Off” errors even though the patches were placed correctly.	5%
Data synchronization issues between the Fitbit App and the data receiving and displaying platform (CGMCare Map)	15%
Difficulty in conducting video conferences with TreC App due to failure to receive notifications.	10%

**Table 4:** Details of the single questions in the satisfaction questionnaire and the related scores.

1. How Do You Judge the System Overall?	2. Was It Easy to Use Devices?	3. Did You Experience Difficulties in Contacting the Service?	4. Were the Indications of the Health Staff Clear?	5. Do You Feel More Secure Since When You Have Access to the Service?	6. Did the Access to the Service Help Your Family or the People You Live with?
4.0 (4.0–4.0)	3.0 (2.0–3.2)	4.0 (4.0–4.0)	4.0 (3.0–4.0)	4.0 (3.0–4.0)	4.0 (3.0–4.0)

## Data Availability

The raw data supporting the conclusions of this article will be made available by the authors at https://www.zenodo.org.

## Abbreviations

The following abbreviations are used in this manuscript:

mHealth	mobile health
SUS	System Usability Scale
CGM	CompuGroupMedical
TreC	web dashboard
